# Polymyxin B and ethylenediaminetetraacetic acid act synergistically against *Pseudomonas aeruginosa* and *Staphylococcus aureus*

**DOI:** 10.1128/spectrum.01709-23

**Published:** 2024-01-03

**Authors:** Samuel J. M. Hale, Alan J. Cameron, Christian A. Lux, Kristi Biswas, Raymond Kim, Mark O'Carroll, Paul W. R. Harris, Richard G. Douglas, Brett Wagner Mackenzie

**Affiliations:** 1 Department of Surgery, Faculty of Medical and Health Sciences, The University of Auckland, Grafton, Auckland, New Zealand; 2 School of Chemical Sciences and School of Biological Sciences, The University of Auckland, Auckland, New Zealand; 3 Maurice Wilkins Centre for Molecular Biodiscovery, The University of Auckland, Auckland, New Zealand; 4 Respiratory Services, Auckland City Hospital, Te Toka Tumai, Te Whatu Ora, Auckland, New Zealand

**Keywords:** biofilms, cystic fibrosis, EDTA, polymyxins, *Pseudomonas aeruginosa*, *Staphylococcus aureus*, synergism

## Abstract

Polymyxin B and ethylenediaminetetraacetic acid are antimicrobials possessing antibiofilm activity. They act by displacement and chelation, respectively, of divalent cations in bacterial membranes and may therefore act synergistically when applied in combination. If so, this combination of agents may be useful for the treatment of diseases like cystic fibrosis (CF), in which biofilms are present on the respiratory epithelium. We used checkerboard assays to investigate the synergy between these agents using reference strains *Pseudomonas aeruginosa* ATCC 27853 and *Staphylococcus aureus* ATCC 6538 in planktonic form. We then determined the efficacy of each agent against biofilms of both species grown on 96-pin lids and proceeded to combination testing against the *P. aeruginosa* reference strain and 10 clinical isolates from patients with CF. Synergism was observed for planktonic forms of both species and for biofilms of *P. aeruginosa*. The susceptibility of biofilms of *P. aeruginosa* clinical isolates to these agents was variable compared to the laboratory reference strain. This combination of agents may be useful in the management of biofilm-associated conditions, particularly those amenable to topical therapies. These results provide a basis upon which the antimicrobial and antibiofilm efficacy of preparations containing these agents may be enhanced.

**IMPORTANCE:**

Bacteria living in biofilms produce a protective matrix which makes them difficult to kill. Patients with severe respiratory disease often have biofilms. Polymyxin B is an antibiotic commonly used in topical medications, such as eye drops and nasal sprays. Ethylenediaminetetraacetic acid (EDTA) is used widely as a preservative in medication but also has antimicrobial properties. It has been hypothesized that Polymyxin B and EDTA could have a synergistic relationship: when used in combination their antimicrobial effect is enhanced. Here, we evaluated the levels at which Polymyxin B and EDTA work together to kill common pathogens *Pseudomonas aeruginosa* and *Staphylococcus aureus*. We found that Polymyxin B and EDTA were synergistic. This synergy may be useful in the management of planktonic infection with *P. aeruginosa* and *S. aureus*, or biofilm infection with *P. aeruginosa*. This synergy may be beneficial in the treatment of respiratory biofilms, in which *P. aeruginosa* biofilms are common.

## INTRODUCTION

Polymyxins are antimicrobial peptides isolated from *Paenibacillus polymyxa* (1). Polymyxin B and colistin (polymyxin E) are used clinically as antibiotics, but their systemic administration carries a high risk of nephrotoxicity and neurotoxicity. Accordingly, they are only used systemically as antibiotics of last resort in critically ill patients infected with multi-resistant organisms (2, 3). However, topical administration does not carry these risks, and polymyxin B is commonly used in antimicrobial preparations such as Maxitrol eye ointment and drops (Novartis Pharma AG, Basel, Switzerland) or Polydexa nasal spray (Rusfic LLC, Moscow, Russia) (4). The mechanism of action of this class is not yet fully understood, although its initial stage involves disruption of the bacterial outer membrane by displacement of divalent cations (Ca^2+^, Mg^2+^) that link membrane lipopolysaccharides (5). The polymyxins are generally considered to be active only against Gram-negative bacteria for this reason, though some studies have reported activity against *Staphylococcus aureus* at higher concentrations (5, 6). Against Gram-positives, polymyxin B is thought to exert its effects by interacting with cell wall teichoic acids, which are likewise stabilized by divalent cations, and by oxidative damage (2). Activity against planktonic cells and Gram-negative biofilms has been described previously (1).

Ethylenediaminetetraacetic acid (EDTA) is a chelating agent that is used widely as a preservative in drug formulations and is also given intravenously for the treatment of heavy metal poisoning (7). EDTA exerts its antibacterial and antibiofilm action by chelating divalent cations in the bacterial membrane and the extracellular polymeric substance of the biofilm, respectively, causing destabilization of these structures (1).

Both polymyxin B and EDTA act by interaction with divalent cations in bacterial membranes, and so it is plausible that they may act synergistically when applied together. Previous studies have demonstrated a potentiating effect of EDTA on the action of polymyxin B against planktonic *Pseudomonas aeruginosa* (8). Others have hypothesized that these agents may have a true synergistic relationship, and while this has not yet been proven for polymyxin B, synergy has recently been demonstrated between EDTA and colistin (polymyxin E) (9, 10). It is, therefore, likely that synergy also occurs between EDTA and polymyxin B, and this may be used to enhance their antimicrobial activity. This is particularly true for infections that are amenable to topical treatment.

Infections that may be treatable with topical therapies include those of the upper and lower airways of patients with cystic fibrosis (CF). Patients with this disease characteristically develop recurrent pulmonary infections and severe chronic rhinosinusitis (CRS), with the presence of *Staphylococcus* and *Pseudomonas* biofilms in the airway being well described and high rates of antibiotic resistance observed (11). Topical therapies are easily applied to the upper respiratory tract in sprays or rinses, and the lower respiratory tract by inhalers or nebulizers. Inhaled polymyxins have been used clinically with beneficial effects (noting a risk of bronchoconstriction due to histamine release), and EDTA is already present as a preservative in common inhaled and nebulized drug preparations (3, 12–14). Furthermore, these agents could be delivered as a nasal spray or in a sinus rinse to manage infection of the paranasal sinuses, which may be a source of seeding to the bronchopulmonary tree (15). Inhaled polymyxins have also been investigated for the treatment of non-CF pulmonary infection in the critically ill, with promising results (16).

We therefore aimed to determine whether polymyxin B and EDTA act synergistically against *P. aeruginosa* ATCC 27853 and *S. aureus* ATCC 6538, growing planktonically and as biofilms. These species were chosen as they are common CF and CRS-associated pathogens. We determined the more general applicability of our results by testing biofilms grown from 10 clinical isolates of *P. aeruginosa* taken from patients with CF, using these agents in combination at the minimum biofilm eradication concentrations (MBECs) previously determined for the reference strain.

## MATERIALS AND METHODS

### Bacterial species

Planktonic testing using polymyxin B and EDTA alone and in combination, and biofilm testing using each agent alone were performed on *S. aureus* ATCC 6538 and *P. aeruginosa* ATCC 27853 strains. Biofilm testing using polymyxin B and EDTA in combination was performed against *P. aeruginosa* ATCC 27853 and 10 clinical isolates of non-mucoid *P. aeruginosa* from five patients with CF (five isolates from sputum and five from middle meatal swabs). When multiple isolates were used from the same patient, these were grown from samples collected at different time points. Hospital laboratory-reported antibiotic susceptibilities of these clinical isolates are reported in Table 1.

**TABLE 1:** Hospital laboratory-reported antibiotic susceptibilities of clinical *Pseudomonas aeruginosa* isolates^*a*^

Isolate	Sample site	Susceptible, standard dosing regimen	Susceptible, increased exposure	Resistant
CF02-SP01	Sputum	Ceftazidime, aztreonam, gentamicin		Amikacin, ciprofloxacin, tobramycin
CF06-SP01	Sputum	Ceftazidime, meropenem, tobramycin		Ciprofloxacin
CF07-SP03	Sputum	Meropenem, tobramycin	Ceftazidime, ciprofloxacin	
CF09-SP02	Sputum	Tobramycin	Ceftazidime	Ciprofloxacin, meropenem
CF12-SP02	Sputum		Ceftazidime, piperacillin-tazobactam	Ciprofloxacin, meropenem, tobramycin
CF02-S04	Sinus	Meropenem, tobramycin	Ceftazidime	Ciprofloxacin
CF07-S01	Sinus	Ceftazidime, ciprofloxacin, meropenem, tobramycin		
CF07-S05	Sinus	Meropenem, tobramycin	Ceftazidime, ciprofloxacin	
CF09-S04	Sinus	Tobramycin		Ceftazidime, ciprofloxacin, meropenem
CF12-S04	Sinus			Ceftazidime, ciprofloxacin, meropenem, tobramycin

^*a*^ Each isolate name denotes patient (CF##), sample site [SP = sputum, S = sinus (middle meatal swab)], and sampling episode. Our hospital laboratory reports a “susceptible, increased exposure” result for *P. aeruginosa* with wild-type sensitivity to the antibiotics in this column.

Stocks of reference strains were sub-cultured using the streak plate method on tryptic soy agar (TSA) (BD Bacto Tryptic Soy Broth; BD Difco Agar, Bacteriological; Becton, Dickinson and Company, Franklin Lakes, NJ, USA). This was incubated overnight at 37°C then stored at 4°C. Single colonies from this plate were used to inoculate ~10 mL of culture media and incubated at 37°C, 200 revolutions per minute (rpm) for overnight broth cultures. Stocks of clinical isolates were stored in tryptic soy broth (TSB) with 50% glycerol at –80°C. These were thawed at room temperature, and 10 µL was used to inoculate ~10 mL of TSB and incubated at 37°C, 200 rpm overnight for overnight broth cultures.

### Patient recruitment, sampling collection, and processing

Adult patients diagnosed with CF and comorbid CRS were recruited from Greenlane Clinical Centre, Auckland, New Zealand commencing 1 September 2020 and finishing 31 August 2022 as part of a prospective, observational longitudinal study. Patients who were pregnant, breastfeeding, or younger than 18 years of age were excluded. Written informed consent from all patients in this study and ethical approval (20/STH/24) from the New Zealand Health and Disability Ethics Committee were obtained. A sterile transport swab for bacterial culture (Transystem, Copan Diagnostics Inc., Murrieta, CA, USA) was collected from the middle meatus on the side with the easiest access to avoid contamination from other sinus sites. The swab sample was placed immediately in a sterile transport medium. An expectorated sputum sample was collected in a sterile pottle. These samples were transferred to Auckland LabPLUS laboratories at Auckland City Hospital, Auckland, New Zealand for routine cultivation and identification. *P. aeruginosa* were identified by colony morphology and confirmed using matrix-assisted laser desorption ionization time-of-flight mass spectrometry (VITEK MS, Biomerieux). Antibiotic resistance and susceptibility profiles for amikacin, aztreonam, gentamicin, ceftazidime, ciprofloxacin, meropenem, and tobramycin were assessed as previously described (17). Isolates were transported to the University of Auckland, New Zealand on TSA slopes and stored at 4°C until each subsample was re-streaked onto TSA plates. These agar plates were incubated overnight at 37°C. A single colony was sub-cultured in sterile TSB for 4 h at 37°C and 200 rpm. Each broth culture was subsampled and stored at a 1:1 vol of 80% glycerol for downstream analyses.

### Materials

Polymyxin B sulfate (I559, AK Scientific, Inc., Union City, CA, USA) was sourced as powder, and solutions were prepared in sterile MilliQ water, 0.9% saline or EDTA solution as appropriate to the experiment. Edetate Disodium USP for injection, 150 mg/mL (Centre for Advanced Medicine Ltd., Auckland, New Zealand) was chosen as an EDTA solution used clinically and was used as a stock solution directly from the ampoule.

Polymyxin B has a high affinity for polystyrene, causing a pseudo-dilution effect when assays are performed on this material (18). For this reason, all testing was performed in polypropylene 96-well plates (Nunc Microwell, part number 267334, Thermo Fisher Scientific, Waltham, MA, USA). Stock solutions were prepared in polypropylene centrifuge tubes (1.5 mL graduated microtubes, part number 1210-07, Scientific Specialties, Inc., Lodi, CA, USA; Falcon 15 mL conical centrifuge tube, part number 352096, Corning Inc., Corning, NY, USA). Samples were handled in polypropylene pipette tips (Neptune Scientific, San Diego, CA, USA). Biofilms were grown on polystyrene 96-pin lids (Nunc Immuno TSP lids, part number 445497, Thermo Fisher Scientific, Waltham, MA, USA), and the surface area of the pins to which the polymyxin B was exposed was kept as close as possible to the surface area containing biofilm; this minimized the degree of polymyxin B binding while ensuring that the entire biofilm-containing surface of the pin was exposed. Biofilm growth, rinsing, and recovery were performed in polystyrene 96-well plates (Nunc Microwell, part number 167008, Thermo Fisher Scientific, Waltham, MA, USA) as polymyxin B was not used for these steps.

Planktonic experiments were performed in Mueller Hinton II broth (MHB) (BBL Mueller Hinton II Broth [cation adjusted]; Becton, Dickinson and Company, Franklin Lakes, NJ, USA) as other culture media adversely affect susceptibility to polymyxin B (18). Biofilms were grown in TSB (BD Bacto Tryptic Soy Broth; Becton, Dickinson and Company, Franklin Lakes, NJ, USA), and antibiofilm testing was performed using antimicrobials in solution with 0.9% sterile saline.

### Planktonic experiments

#### Minimum inhibitory and bactericidal concentrations

Minimum inhibitory concentration (MIC) and minimum bactericidal concentration (MBC) assays were performed by the broth microdilution technique according to Wiegand et al. (19) and Moody and Knapp (20), respectively, with modifications. Briefly, a stock solution of polymyxin B was prepared in sterile MilliQ water and mixed in equal parts with double (2×) concentrated MHB. A solution of 135 mg/mL EDTA in MHB was prepared by adding 9 parts EDTA solution from the ampoule to 1 part 10× concentrated MHB. Two hundred microliters of these solutions was then added to wells in the top row of polypropylene 96-well plates. A doubling dilution series was performed in MHB leaving 100 µL in each well. Overnight cultures of the test organisms were adjusted to 1 × 10^8^ colony forming units (CFU)/mL by optical density at 600 nm (OD600) using a spectrophotometer (Libra S22, Biochrom US, Holliston, MA, USA), and 100 µL was added to each well to give a final inoculum of 5 × 10^7^ CFU/mL. This was incubated overnight at 37°C at 200 rpm in an orbital shaking incubator.

The following day, 95 µL from each well was transferred to a clear flat-bottom polystyrene 96-well plate (Nunc Microwell, part number 167008, Thermo Fisher Scientific, Waltham, MA, USA) for measurement of OD600 (EnSight multimode plate reader, PerkinElmer, Inc., Waltham, MA, USA). The lowest concentration of antimicrobial at which no increase in OD600 was observed was determined to be the MIC. A further 10 µL from each well was spot-plated on TSA and incubated overnight at 37°C. The lowest concentration of antimicrobial at which fewer than 25 colonies grew was determined to be the MBC, consistent with an approximate 5log_10_ reduction in viable bacteria.

### Checkerboard assays

Stock solutions of quadruple concentration (4×) polymyxin B were prepared in sterile MilliQ water and 150 mg/mL EDTA. Two hundred microliters of 4× polymyxin B in EDTA was added to the top left well (A1) of a 96-well plate with 200 µL of double concentration (2×) MHB. One hundred microliters of 4× polymyxin B in MilliQ was added to the remaining wells in the left-most column (B1–H1), and 100 µL of 150 mg/mL EDTA was added to the next nine wells in the top row (A2–A10) with 100 µL of 2× MHB. One hundred microliters of MHB was added to the remaining empty wells from B2 to H10. Columns 11 and 12 were used for sterility (sterile MHB) and growth controls (untreated bacterial inoculum). Doubling dilutions were performed from row A to row G, then from column 1 to column 9. This created a checkerboard with combinations of polymyxin B and EDTA in wells A1 to G9, a doubling dilution series of polymyxin B alone in row H, and a doubling dilution series of EDTA alone in column 10. Overnight cultures were prepared by the same method as for MIC/MBC assays, and 100 µL of inoculum was added to each well. Incubation, OD600 measurement, spot plating, and colony counting were performed as for MIC/MBC assays.

Synergy was determined by calculating the fractional inhibitory concentration index and fractional bactericidal concentration index (FICI and FBCI), using the following formulae:  


$$\begin{array}{l} FICI=PMB\;MIC\;(combination)/PMB\;MIC\;(alone) \end{array}\;\begin{array}{c} +EDTA\;MIC\;(combination)/EDTA\;MIC\;(alone), \end{array}$$

$$\begin{array}{l} FBCI=PMB\;MBC\;(combination)/PMB\;MBC\;(alone) \end{array}\;\begin{array}{c} +EDTA\;MBC\;(combination)/EDTA\;MBC\;(alone). \end{array}$$

An index value ≤0.5 indicates synergy between the tested compounds, with values between 0.5 and 4 indicating additivity or indifference, and >4 indicating antagonism (21, 22). The antimicrobial combinations with the lowest FICI and FBCI in each checkerboard were recorded.

### Biofilm experiments

#### MBEC assays

Biofilms were grown on 96-pin lids (Nunc Immuno TSP lids, part number 445497, Thermo Fisher Scientific, Waltham, MA, USA) using a modified version of a previously described protocol (23). Briefly, overnight cultures were diluted to ~10^7^ CFU/mL in TSB and 150 µL added to the wells of a 96-well plate (Nunc Microwell, part number 167008, Thermo Fisher Scientific, Waltham, MA, USA). TSB alone was added to four wells for sterility control. The 96-pin lid was then placed and incubated at 37°C in an orbital shaking incubator for 24 hours, at 125 rpm for *P. aeruginosa* and 150 rpm for *S. aureus*.

Stock solutions of polymyxin B were prepared in sterile 0.9% saline, and a 135 mg/mL EDTA and 0.9% saline solution was prepared by combining 9 parts 150 mg/mL EDTA solution with 1 part 9% saline in order to maintain stable tonicity throughout the dilution series. Four hundred microliters of the stock solutions was added to the wells of the top row of 96-well polypropylene plates in portrait orientation. A doubling dilution series was performed in 0.9% saline, leaving 200 µL of treatment solution in each well. The 96-pin lids carrying biofilm were transferred to this 96-well plate and incubated in static conditions at room temperature for 24 hours.

After treatment, pins were rinsed twice by transferring them to two new 96-well plates containing 200 µL sterile 0.9% saline in each well, then transferred to a final 96-well plate containing 200 µL sterile 0.9% saline in each well for recovery. This 96-well plate was placed in a polypropylene tray and sonicated at 37 kHz for a total of 30 minutes. Ice was placed in the ultrasonic bath (ELP030H; Elma Schmidbauer GmbH, Singen, Germany) at the start and replaced at 15 minutes to prevent undue heating. Ten microliters from each well was spot-plated on TSA and incubated at 37°C overnight. The lowest concentration at which five or fewer colonies grew following treatment, and recovery was determined to be the minimum biofilm eradication concentration.

Biofilms were also grown and recovered immediately without treatment to determine bacterial counts at baseline. After sonication, a 10-fold dilution series was performed in sterile 0.9% saline, and 10 µL of each dilution was spot-plated on TSA and incubated as above. Colonies were counted and bacterial counts per pin were determined by back-calculation.

#### Combination testing

Following determination of the FBCI and MBECs for *P. aeruginosa*, combination testing was performed against *P. aeruginosa* ATCC 27853. A doubling dilution series was set up with polymyxin B and EDTA in the concentration ratio expected to demonstrate synergy against biofilm, based on the results of the preceding planktonic and biofilm experiments. Biofilms were grown, treated, and recovered as previously described. FBCI for biofilm (FBCI_b_) was calculated by using the MBEC of each antimicrobial in combination and alone, as for FICI and FBCI for planktonic experiments.

#### Clinical isolate testing

Biofilms of clinical isolates of *P. aeruginosa* were grown on 96-pin lids as described above. For biofilm growth, the OD600 of the inoculum for each clinical isolate was adjusted with TSB to the OD600 of the *P. aeruginosa* ATCC 27853 inoculum, ensuring uniform inoculation despite variation in overnight cultures between strains. Polypropylene 96-well plates were prepared with 200 µL of a polymyxin B + EDTA + 0.9% saline solution per well, at the MBEC determined in the previous step for these agents in combination. Treatment and recovery were performed as previously described. Furthermore, 10 µL from each well of the plate containing treatment solutions in which pins had been immersed was spot-plated on TSA to ensure that the loss of viable cells was truly due to bacterial eradication and not simply dispersal into solution. Biofilms from which five or fewer colonies grew following treatment and recovery were deemed to be eradicated.

### Scanning electron microscopy

Scanning electron microscopy (SEM) was undertaken to provide evidence of biofilm formation from all bacterial isolates tested in this experiment. Pins carrying biofilm from each strain were broken off the 96-pin lid and fixed in 2.5% glutaraldehyde, rinsed in phosphate-buffered saline, and dehydrated by serial immersion in increasing concentrations of ethanol. Samples were then affixed to aluminum stubs and gold sputter-coated (DSR1, Nanostructured Coatings Co., Tehran, Iran) before undergoing SEM (TM3030Plus, Hitachi Ltd., Tokyo, Japan).

### Statistics

All experiments were performed in biological duplicate. MIC, MBC, FICI, FBCI, MBEC, and FBCI_b_ are expressed as median ± range. Where the median concentration fell between two values, the higher of these values was reported. Replicates for which MBECs could not be reliably determined were excluded. Statistical analyses were conducted in R version 4.1.1 (24). Mann-Whitney *U* test was used to compare MIC, MBC, and MBEC data. Fisher’s exact test was used to compare susceptibility of *P. aeruginosa* clinical isolates to the laboratory reference strain.

## RESULTS

### Planktonic experiments

In planktonic testing with reference strains, *P. aeruginosa* ATCC 27853 was observed to be significantly more susceptible to polymyxin B than *S. aureus* ATCC 6538 (*P* < 0.002) in keeping with the known spectrum of activity of this antibiotic. *S. aureus* was significantly more susceptible to EDTA than *P. aeruginosa* (*P* < 0.002). MIC and MBC data for *S. aureus* and *P. aeruginosa* are summarized in Table 2. When tested in combination, synergy was observed between polymyxin B and EDTA toward planktonic forms of both species. Against *S. aureus,* FICI was 0.5 (0.31–0.75) (median [range]) though FBCI was slightly greater than the cutoff for synergy at 0.56 (0.52–1.0). Against *P. aeruginosa*, synergy was clearly demonstrated for both growth inhibition (FICI 0.39 [0.26–0.39]) and bactericide (FBCI 0.39 [0.32–0.53]) (Tables 2 and 3).

**TABLE 2:** MIC and FICI values against planktonic *Staphylococcus aureus* ATCC 6538 and *Pseudomonas aeruginosa* ATCC 27853^*a*^

	PMB MIC_a_ (µg/mL)	PMB MIC_c_ (µg/mL)	*P* value (Mann-Whitney *U*)	EDTA MIC_a_ (mg/mL)	EDTA MIC_c_ (mg/mL)	*P* value (Mann-Whitney *U*)	FICI
*S. aureus*	256	64 (8–64)	0.002	4.2 (4.2–8.4)	1.2 (1.2–2.3)	0.002	0.5 (0.31–0.75)
*P. aeruginosa*	4	0.5	0.0009	67.5	9.4 (4.7–9.4)	0.001	0.39 (0.26–0.39)

^*a*^ All values are expressed as median (range). If there was no variation between replicates, no range is given. Where the median fell between two values, the higher of these values was recorded. PMB: polymyxin B. MIC_a_: minimum inhibitory concentration of the antimicrobial when tested alone, recorded from MIC assays. MIC_c_: minimum inhibitory concentration of the antimicrobial when tested in combination, recorded from checkerboard assays. FICI was calculated using MIC_a_ from the checkerboard assays except where the MIC_a_ determined in MIC assays was greater than that which could be tested on the checkerboard. Six technical replicates were performed per species.

**TABLE 3:** MBC and FBCI values against planktonic *Staphylococcus aureus* ATCC 6538 and *Pseudomonas aeruginosa* ATCC 27853^*a*^

	PMB MBC_a_ (µg/mL)	PMB MBC_c_ (µg/mL)	*P* value (Mann-Whitney *U*)	EDTA MBC_a_ (mg/mL)	EDTA MBC_c_ (mg/mL)	*P* value (Mann-Whitney *U*)	FBCI
*S. aureus*	512 (512–2,048)	256 (8–256)	0.003	16.9	4.7 (0.6–9.4)	0.002	0.56 (0.52–1.0)
*P. aeruginosa*	4	1 (0.5–2)	0.002	67.5	9.4 (0.6–18.8)	0.002	0.39 (0.32–0.53)

^*a*^ All values are expressed as median (range). If there was no variation between replicates, no range is given. Where the median fell between two values, the higher of these values was reported. PMB: polymyxin B. MBC_a_: minimum bactericidal concentration of the antimicrobial when tested alone, reported from MBC assays. MBC_c_: minimum bactericidal concentration of the antimicrobial when tested in combination, reported from checkerboard assays. FBCI was calculated using MBC_a_ from the checkerboard assays except where the MBC_a_ determined in MBC assays was greater than that which could be tested on the checkerboard. Six technical replicates were performed per species.

### Biofilm experiments

#### MBEC assays

In biofilm testing, polymyxin B had an MBEC of 32 µg/mL against *P. aeruginosa* and EDTA had an MBEC of 135 mg/mL. In combination, median MBEC was 8 µg/mL for polymyxin B and 16.9 mg/mL for EDTA (*P* < 0.0001 compared to MBECs when tested alone). This gave an FBCI_b_ of 0.38. Against *S. aureus*, polymyxin B had an MBEC of 16,384 µg/mL; however, viable bacteria were consistently recovered even at the highest tested concentration of EDTA. The MBEC for EDTA could, therefore, not be determined, and combination testing was not performed (Table 4).

**TABLE 4:** Minimum biofilm eradication concentration and fractional bactericidal concentration index against biofilm (FBCI_b_) values of polymyxin B and EDTA, alone and in combination against biofilms of *Staphylococcus aureus* ATCC 6538 and *Pseudomonas aeruginosa* ATCC 27853^*a*^

	PMB MBEC_a_ (µg/mL) (*n*)	PMB MBEC_c_ (µg/mL) (*n*)	*P* value	EDTA MBEC_a_ (mg/mL) (*n*)	EDTA MBEC_c_ (mg/mL) (*n*)	*P* value	FBCI_b_
*S. aureus*	16,384 (4,096–32,768) (15)			>135 (67.5 to >135) (11)			
*P. aeruginosa*	32 (8–64) (16)	8 (4–32) (16)	<0.0001	135 (13)	16.9 (8.4–67.5) (16)	<0.0001	0.38 (0.19–1.1)

^*a*^ All values are expressed as median (range). If there was no variation between replicates, no range is given. *P* values were determined by the Mann-Whitney *U* test. PMB: polymyxin B. MBEC_a_: minimum biofilm eradication concentration of the antimicrobial tested alone. MBEC_c_: minimum biofilm eradication concentration of the antimicrobial tested in combination. *n*: number of replicates.

At baseline, *P. aeruginosa* biofilms contained a median of 8.0 × 10^6^ (2.0 × 10^6^ to 1.4 x 10^8^) CFU/pin, and *S. aureus* biofilms contained a median of 6.0 × 10^5^ (8.0 ×10^4^ to 1.4 × 10^6^) CFU/pin. The limit of detection for this assay is 20 CFU/pin, or 1.3log_10_. Accordingly, these MBECs approximate a 5.5log_10_ reduction for *P. aeruginosa* and 4.4log_10_ reduction for *S. aureus*, reflecting differences between these species in this model of biofilm growth.

#### Clinical isolates

Of the 10 *P. aeruginosa* clinical isolates tested against polymyxin B and EDTA in combination (8 µg/mL and 16.9 mg/mL, respectively), three were completely or almost completely eradicated, three were eradicated in 2/3 of replicates or more, three were eradicated in fewer than 1/4 of replicates, and one was not eradicated at all. These results highlight the heterogeneity in susceptibility to antimicrobial agents between clinical isolates. When the laboratory reference strain was tested alongside the clinical isolates, biofilm was eradicated in just under half of all replicates, in keeping with the range of concentrations leading to eradication in the formal MBEC assays. Of 183 pins from which biofilms were eradicated, no viable bacteria were recovered from the corresponding treatment solution in 180 replicates indicating that biofilm eradication was due to bacterial killing and not dispersal into solution.

#### Scanning electron microscopy

**Fig 1:**
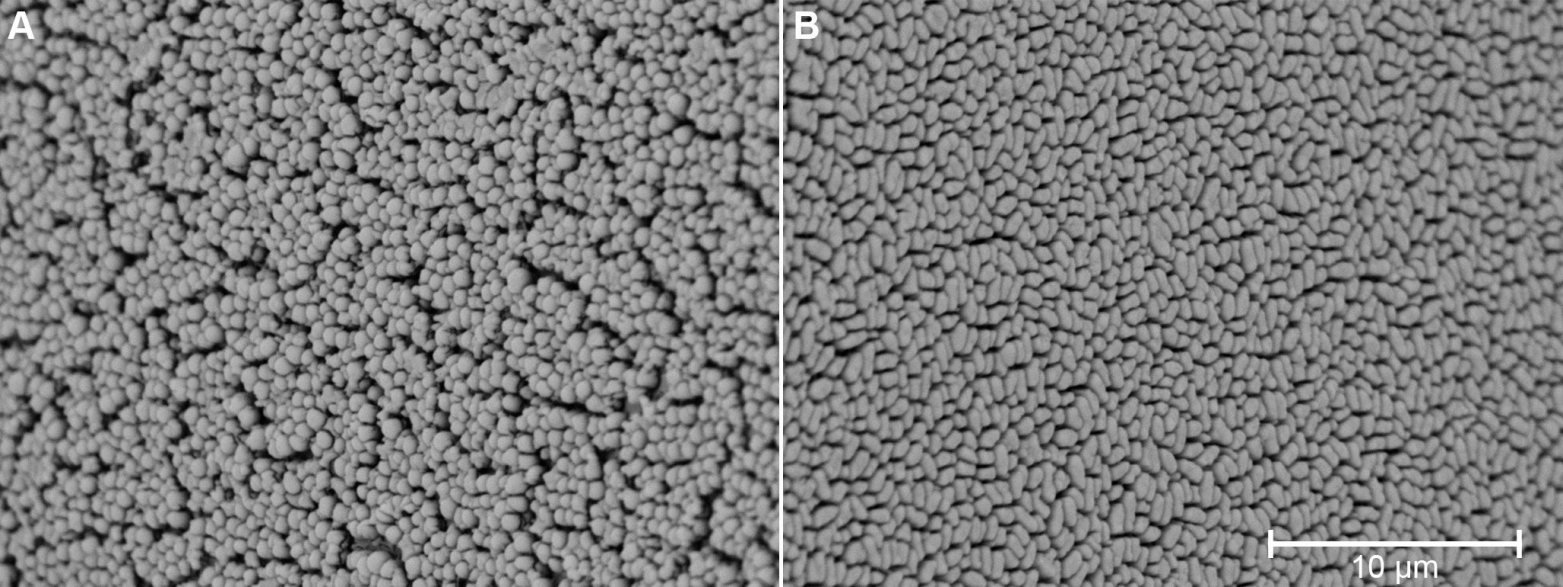
Scanning electron micrographs (8,000x magnification) of type strains grown as biofilms. (A) *Staphylococcus aureus* ATCC 6538 and (B) *Pseudomonas aeruginosa* ATCC 27853.

**Fig 2:**
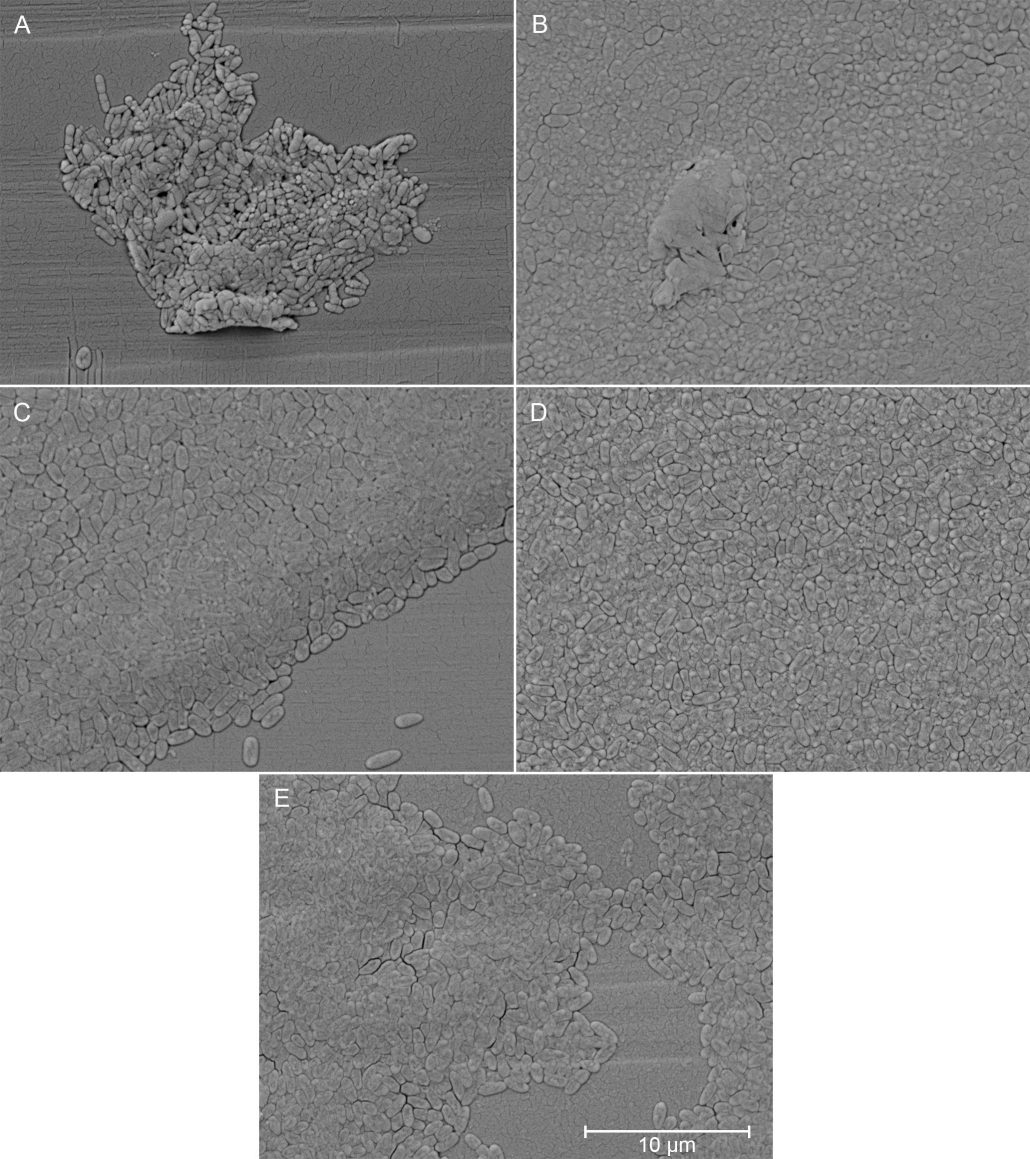
Scanning electron micrographs (8,000x magnification) of clinical isolates from sputum grown as biofilms. (A) CF02-SP01, (B) CF06-SP01, (C) CF07-SP03, (D) CF09-SP02, and (E) CF12-SP02.

**Fig 3:**
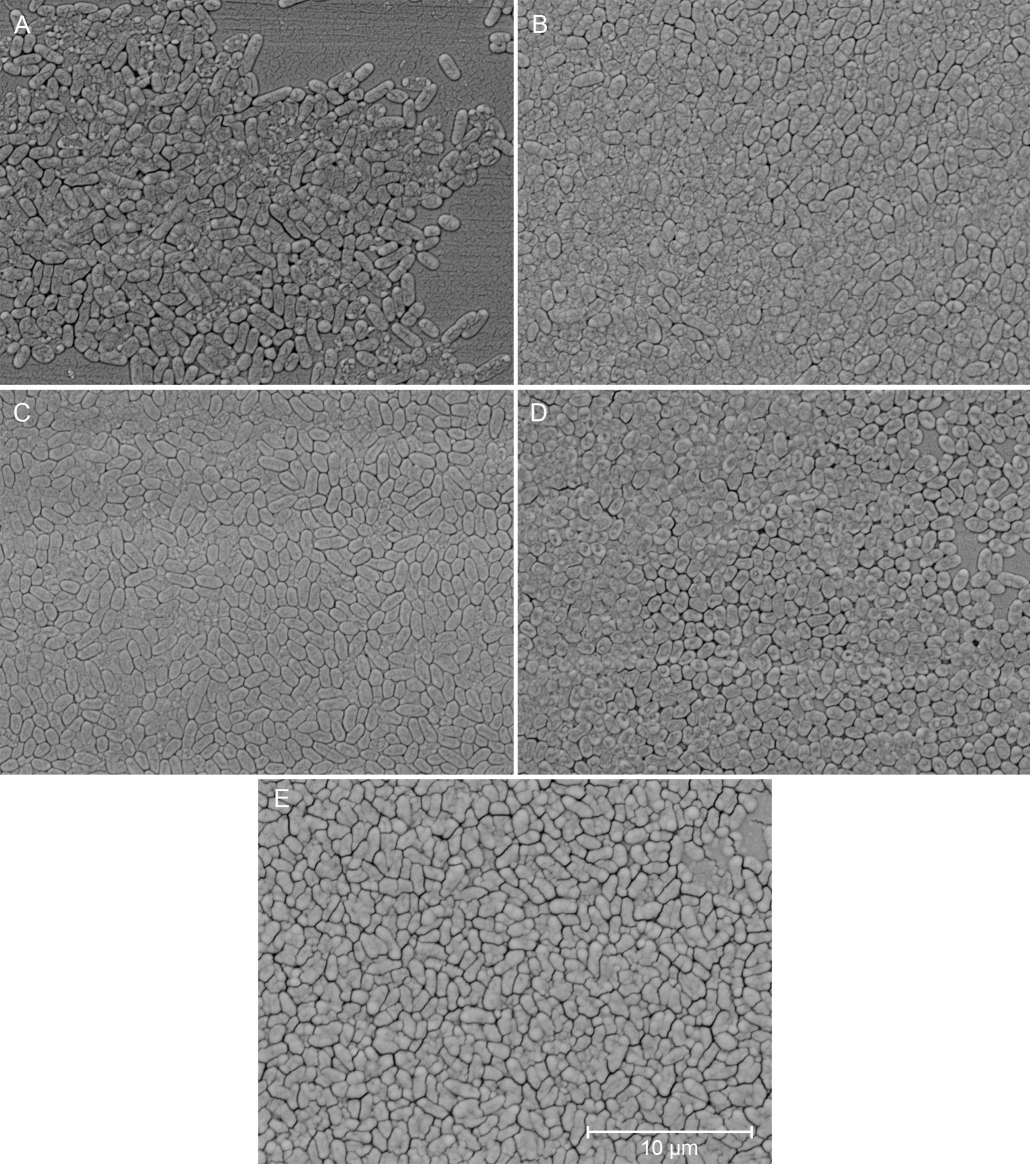
Scanning electron micrographs (8,000x magnification) of clinical isolates from middle meatal swabs grown as biofilms. (A) CF02-S04, (B) CF07-S01, (C) CF07-S05, (D) CF09-S04, and (E) CF12-S04.

Biofilm growth was observed across all isolates on pin lids using SEM (Fig. 1 to 3).

## DISCUSSION

In our study, marked reductions were observed in the minimum effective concentrations of both agents when applied together against both tested species’ planktonic forms, and in the MBEC for *P. aeruginosa* ATCC 27853. The MBEC of EDTA was greater than the highest tested concentration for *S. aureus* ATCC 6538, so FBCI_b_ could not be determined and the MBEC of polymyxin B was far higher than any clinically achievable concentration. Combination testing was, therefore, not performed for *in vitro* biofilms of *S. aureus*.

The MIC and MBC of polymyxin B for *S. aureus* are significantly higher than for *P. aeruginosa*, in keeping with known differences in susceptibility of these organisms to this antibiotic. Synergy was more clearly observed against *P. aeruginosa* than *S. aureus*, with the observed FICI, FBCI, and FBCI_b_ all being less than 0.5. Against *S. aureus*, FICI was 0.5 and FBCI was 0.56, this latter value slightly greater than that suggesting synergy has occurred. Synergy for inhibition of growth but not bacterial killing of *S. aureus* may relate to differences in mechanisms of action for Gram-positive compared to Gram-negative organisms.

A broader range of FICI and FBCI values was observed across replicates for *S. aureus* than *P. aeruginosa*. Furthermore, variability in the susceptibility of *P. aeruginosa* biofilms grown from clinical isolates was observed compared to the reference strain. This suggests that while testing against the reference strain offers helpful insights, testing against clinical isolates remains of value in assessing real-world applicability. Previous studies that have used these methodologies have also observed such variability. One study investigated synergy between polymyxin B and gramicidin S and identified significant variation in FICI between and within 28 strains of *P. aeruginosa* (22). Another investigated synergy between polymyxin B and doxycycline and observed variation of FICI between 0.5 and 1 among 18 *P*. *aeruginosa* clinical isolates (21). We observed no differences in patterns of biofilm eradication by sampling site of origin of the clinical isolate, nor by susceptibility to other antibiotics.

### Previous studies support the use of polymyxins and EDTA for biofilm eradication

#### Polymyxins

Previously, colistin (polymyxin E) and EDTA have been demonstrated to act synergistically against *Klebsiella pneumoniae* clinical isolates*,* both in planktonic form and as biofilms grown in 96-well plates. This combination of agents eradicated biofilms despite the tested strains being colistin-resistant, suggesting that such a combination may have specific merits in the context of the global crisis of antimicrobial resistance. Furthermore, colistin and EDTA treatment reduced bacterial load and improved survival in a mouse model of infection with these organisms, when compared to monotherapy with either agent (10, 25).

Other researchers have tested colistin up to 1,024 µg/mL against biofilms of *S. aureus* (MRSA ATCC 43300) grown in 96-well plates. MBEC was not determined, in keeping with our finding that polymyxin B eradicated *S. aureus* biofilm at much higher concentrations (26). Other authors grew *P. aeruginosa* PA01 biofilms for 3 days in microtitre plates and demonstrated an approximately 5log_10_ reduction in viable bacteria with treatment with 32 µg/mL colistin for 24 hours. However, in anaerobic conditions, an approximately 7log_10_ reduction was seen with 16 µg/mL colistin. This is relevant to CF where airway mucus and immune cells may create hypoxic niches in which *P. aeruginosa* biofilms may develop (27).

Synergy between polymyxin B and other agents has been previously demonstrated. When applied with gramicidin S, polymyxin B is thought to permeabilize the outer membrane of Gram-negative pathogens, allowing gramicidin S to access its site of action at the inner membrane. This combination also yielded an enhanced antibiofilm effect (22). Synergy has also been observed between polymyxin B and doxycycline against *P. aeruginosa in vitro*, with potential clinical benefit subsequently demonstrated in a murine model of *P. aeruginosa* pneumonia. Mice were treated 12-hourly with nebulized polymyxin B (7.5 mg/kg/day), doxycycline (1.28 g/kg/day) or both, beginning 12 hours post-infection for a total of four doses. After euthanasia at 60 hours post-infection, lung tissue was homogenized and cultured. A reduction in viable *P. aeruginosa* of approximately 1log_10_ cells/g of lung tissue was observed with single-agent treatment, but 3log_10_ cells/g with dual therapy (21).

Bacterial biofilms are recognized to have innately reduced antibiotic susceptibility, with tolerance to antibiotics up to 1,000 times the MIC for planktonic forms of the same strain. This is thought to be multifactorial in mechanism, with contributions from restriction of diffusion of antimicrobial compounds through the biofilm, altered bacterial metabolism and formation of persister cells, and the profoundly hypoxic and acidic milieu found deep within the biofilm (28). In contrast to many antibiotics, the polymyxins act primarily by physical disruption of the bacterium, with interference with cellular metabolic processes playing a lesser role (5). This provides a likely explanation as to why substantial biofilm eradication is observed in this study and in the studies cited above, and at comparably modest increases in concentration from the planktonic MIC.

#### EDTA

The antibiofilm activity of EDTA has been widely recognized. In one study, tetrasodium EDTA was tested against biofilms grown on 96-pin lids of clinical isolates of several species from central venous access devices and blood cultures. MBECs at 24 hours were 40 mg/mL for *P. aeruginosa* and 5–40 mg/mL across three different *S. aureus* isolates (29). Another study tested 40 mg/mL tetrasodium EDTA against biofilms grown in the same model and observed complete eradication of *S. aureus* and *Staphylococcus epidermidis* biofilm after 24 hours exposure and a 5–6log_10_ reduction in *P. aeruginosa*, though eradication for this organism was incomplete (30). This contrasts with the present study in which *S. aureus* biofilm was not consistently eradicated even at 135 mg/mL EDTA. This may reflect differences between strains or the EDTA preparation used. Tetrasodium EDTA solutions are basic with a pH greater than 9. In our study, the EDTA preparation chosen was a disodium EDTA solution (acidic) that was pH corrected during manufacturing to between 6.5 and 7.5 using sodium hydroxide. It therefore contains a combination of disodium and trisodium salts.

EDTA has also been recognized to potentiate the antibiofilm effects of other compounds. One group tested EDTA in combination with several antiseptics on biofilms of strains sourced from Collection de l’Institut Pasteur Paris (CIP), *S. aureus* CIP 4.83, and *P. aeruginosa* CIP 103.467, grown on glass coverslips. A 200-fold increase in antibiofilm activity was reported when 20 mmol/L (approximately 6 mg/mL) EDTA was applied with Betadine (10% povidone-iodine, Meda Pharma, Solna, Sweden) compared to Betadine alone; 4,000-fold when applied with Prontosan (0.1% polyhexamethylene biguanide and 0.1% betaine, B. Braun, Melsungen, Germany), and 20,000-fold when applied with Octenilin (0.05% octenidine dihydrochloride, Schülke & Mayr GmbH, Norderstedt, Germany) (31). Antibiofilm efficacy was reported using different measures in the present study, so direct comparison is difficult. This potentiation may, however, be partly due to a dispersal effect on the intact biofilm: another group used a flow-cell model to demonstrate an increase in *P. aeruginosa* cellular detachment from the biofilm upon exposure to EDTA (32).

### Synergistic effect of polymyxin B and EDTA holds promise for clinical application in biofilm-related disease

The role of bacterial biofilms in human disease is increasingly recognized, but few effective agents are available for application to the upper or lower airways. Investigations into the antibiofilm efficacy of the polymyxins are of interest as the polymyxins are already in use in the setting of airway infection.

*P. aeruginosa* clinical isolates in the current study were taken from patients with CF due to their high rates of colonization with this organism, as well as the high prevalence of antibiotic resistance observed in this group (Table 1) (11). Polymyxins, chiefly colistin sulfomethate, have been used as inhaled antibiotics for the treatment of both CF and non-CF bronchopulmonary infections (5, 16). Intravenous polymyxins may also be used in these settings as antibiotics of last resort (3, 5). Methods by which their efficacy may be enhanced are, therefore, of great clinical interest.

In CF treatment, nebulized colistin is preferred over polymyxin B because of its lower risk of bronchoconstriction, due to its lesser propensity for inducing histamine release (3). Bronchoconstriction remains a common adverse effect with colistin, with a decrease in forced expiratory volume in 1 second (FEV_1_) of greater than 10% in one-third of children and smaller symptomatic reductions in FEV_1_ in adults (12, 13). However, topical polymyxin B is commonly applied to mucosal surfaces outside of the bronchopulmonary tree, including the sinonasal mucosa and conjunctiva. EDTA is also commonly applied to mucosal surfaces and is present in inhaled pharmaceutical preparations as a preservative. The maximum safe inhaled dose has yet to be determined: no significant reduction in pulmonary function was observed after a total dose of 2.4 mg nebulized EDTA (four doses of 3 mL of 200 µg/mL solution) in one study though a previous study had demonstrated a 20% reduction in FEV1 after exposure to 2.4 mg/mL nebulized EDTA (total dose unclear) (14, 33). Further clarification of the limits of safety is required prior to application of higher doses of EDTA to the lower airways. Although outside the scope of this study, both safety and the potential for clinical efficacy should be investigated using an animal model of airway infection before contemplating human trials.

### Limitations and strengths

It is recognized that different models of biofilm growth produce biofilms of varying susceptibility to antibiofilm treatments. Biofilms grown on 96-pin lids are less robust than models such as the Centers for Disease Control and Prevention (CDC) Biofilm Reactor. However, pin lids allow for antibiofilm testing with much smaller quantities of treatment solution. Antibiofilm testing using the CDC biofilm reactor would be impractical for this work due to the quantities of antibiotic that would be required for MBEC determination. More broadly, current research in polymyxins includes the development of novel polymyxins and analogues with greater potency and lower toxicity (34, 35). While commercial polymyxin B and EDTA are available in large quantities and could be tested using the CDC biofilm reactor, any future testing of novel polymyxins and analogues produced by necessity at small scale would be unachievable using that methodology (35). Previous researchers testing novel polymyxins have used 96-pin lids for biofilm testing (34). Using this model makes the results of this work more comparable with previous work. Furthermore, the biofilm reactor produces extremely robust biofilms in ideal conditions not replicated *in vivo*, making 96-pin lids an appropriate choice of model for this study. Multiple biofilms of different species or polymicrobial biofilms are likely to be present in diseases like CF. Testing against single-organism biofilms of a wider range of species and against polymicrobial biofilms may offer valuable insights before progressing to animal and human trials.

The great strength of our study is the sequential order in which we determined the MIC, MBC, FICI, FBCI, and FBCI_b_. This experimental design provides a framework for testing of antimicrobial synergy against bacterial biofilms that can be easily replicated by other researchers. SEM was used to demonstrate that all tested strains produced biofilm in these experimental conditions, enhancing methodological rigor. We used both type strains and clinical isolates to illustrate the potential real-world applicability of these results. In addition, we describe an efficient methodology for preparing checkerboard assays. This is similar to a previously published protocol but allows for testing where one antimicrobial is a liquid rather than a powder from which a stock solution can be made (36).

### Conclusion

We have demonstrated synergy between polymyxin B and EDTA against bacteria. These agents are common constituents of topical preparations, and this synergy may be clinically useful in the management of planktonic infection with *P. aeruginosa* and *S. aureus* or biofilm infection with *P. aeruginosa*. This synergy may be beneficial in the treatment of respiratory biofilms in diseases such as CF, in which *P. aeruginosa* biofilms are common. This combination of agents is likely to be safe to apply to the upper airways although further safety and tolerability studies are required, particularly prior to investigating the application of polymyxin B and EDTA to the lower respiratory tract.
